# Supplementary figures and images for: Cyclin-dependent kinase inhibitor 1 plays a more prominent role than activating transcription factor 4 or the p53 tumour suppressor in thapsigargin-induced G1 arrest
Source: PeerJ. 2023 Dec 18;11:e16683. doi: 10.7717/peerj.16683 (PMC10734451; doi:10.7717/peerj.16683)

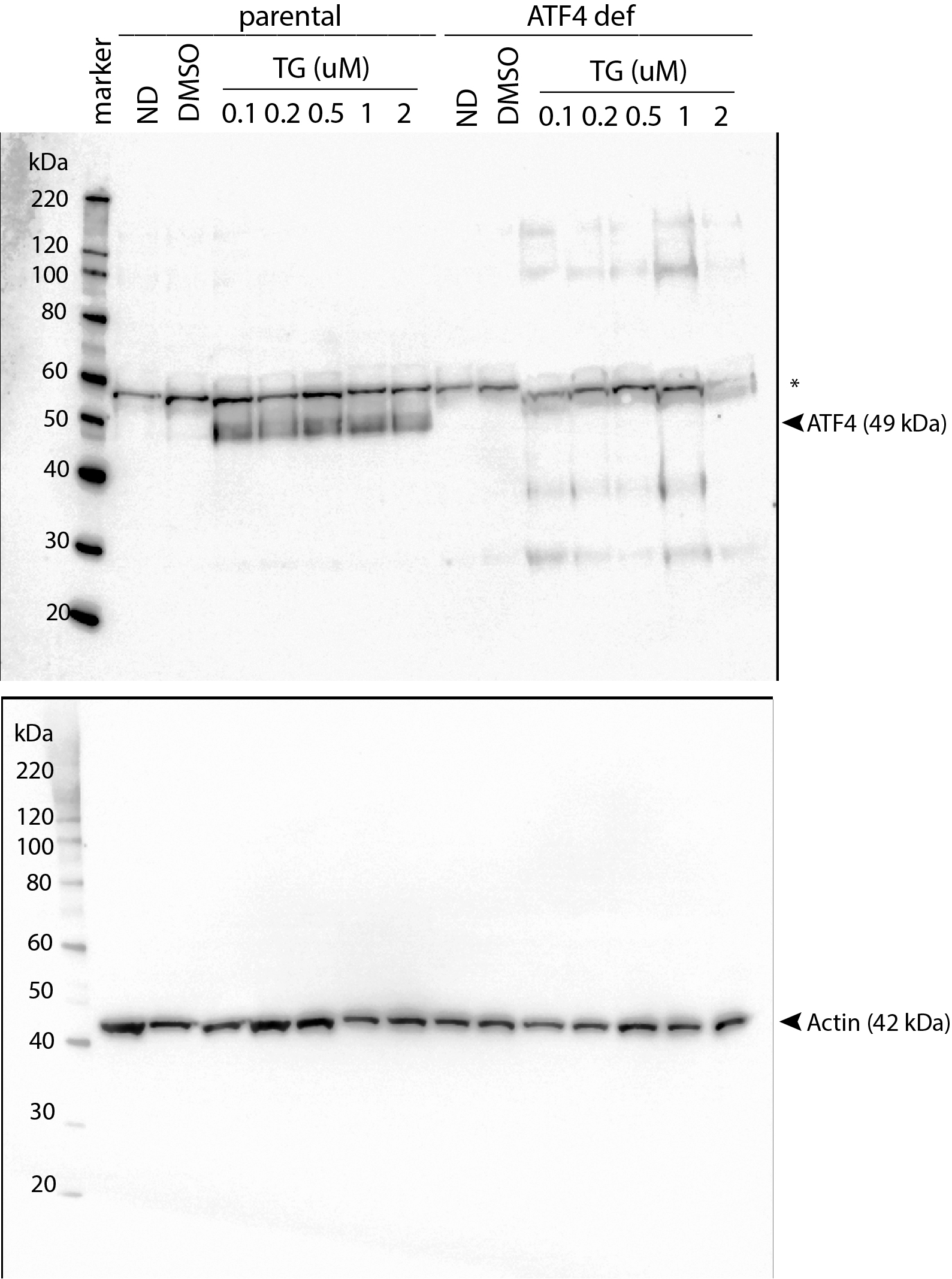

Supplement: Figure S1 — HCT116 and HCT116 ATF4 deficient cells were treated with 0.1, 0.2, 0.5, 1 and 2 µM Tg for 24 h. Protein was extracted and immunoblot analysis of ATF4 and β-actin levels was performed. [file peerj-11-16683-s001.jpg]

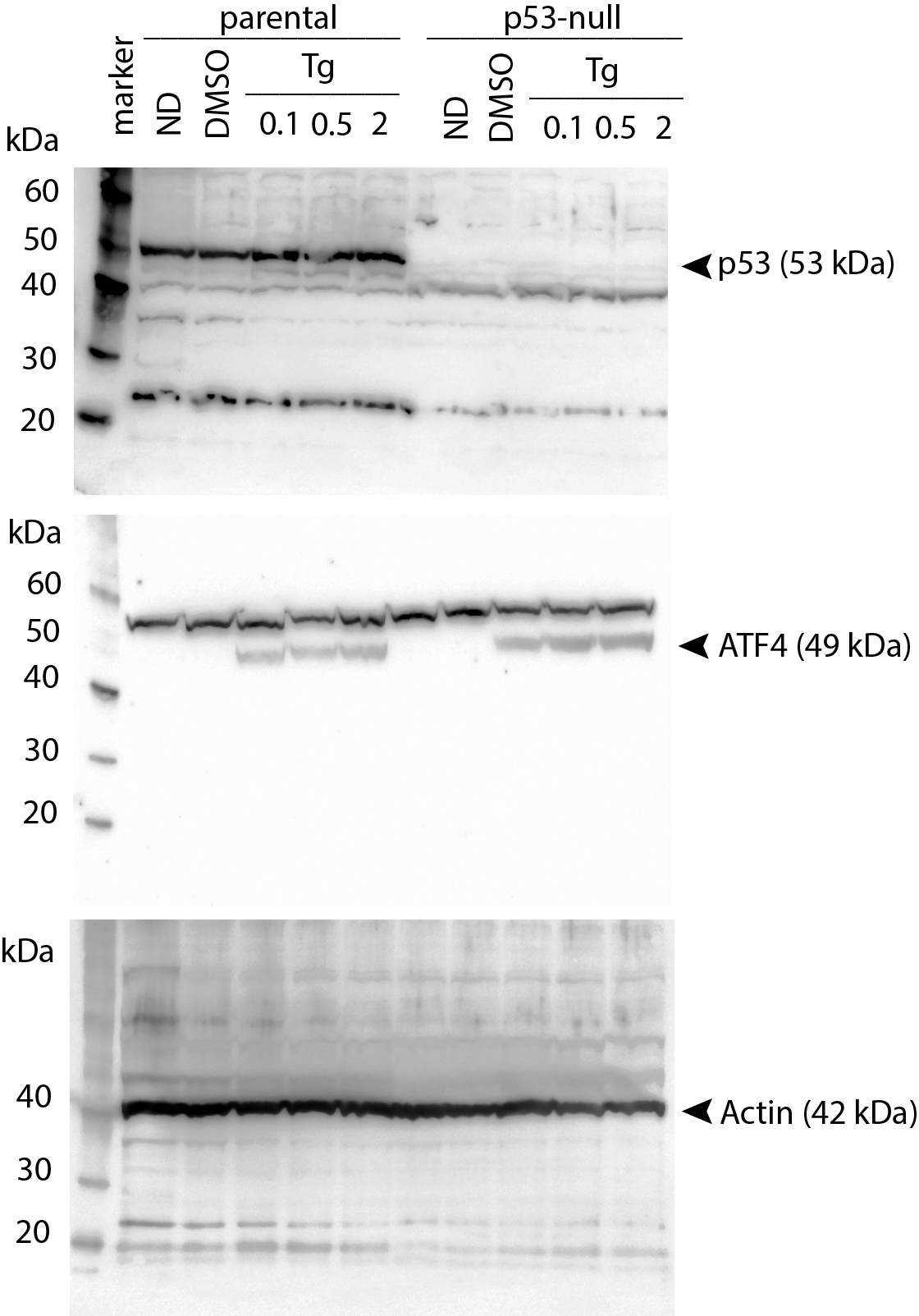

Supplement: Figure S2 — HCT116 and HCT116 p53 −/ − cells were treated with 0.1, 0.2, 0.5, 1 and 2 µM Tg 24 h. Protein was extracted and immunoblot analysis of p53, ATF4 and β-actin was performed. [file peerj-11-16683-s002.jpg]

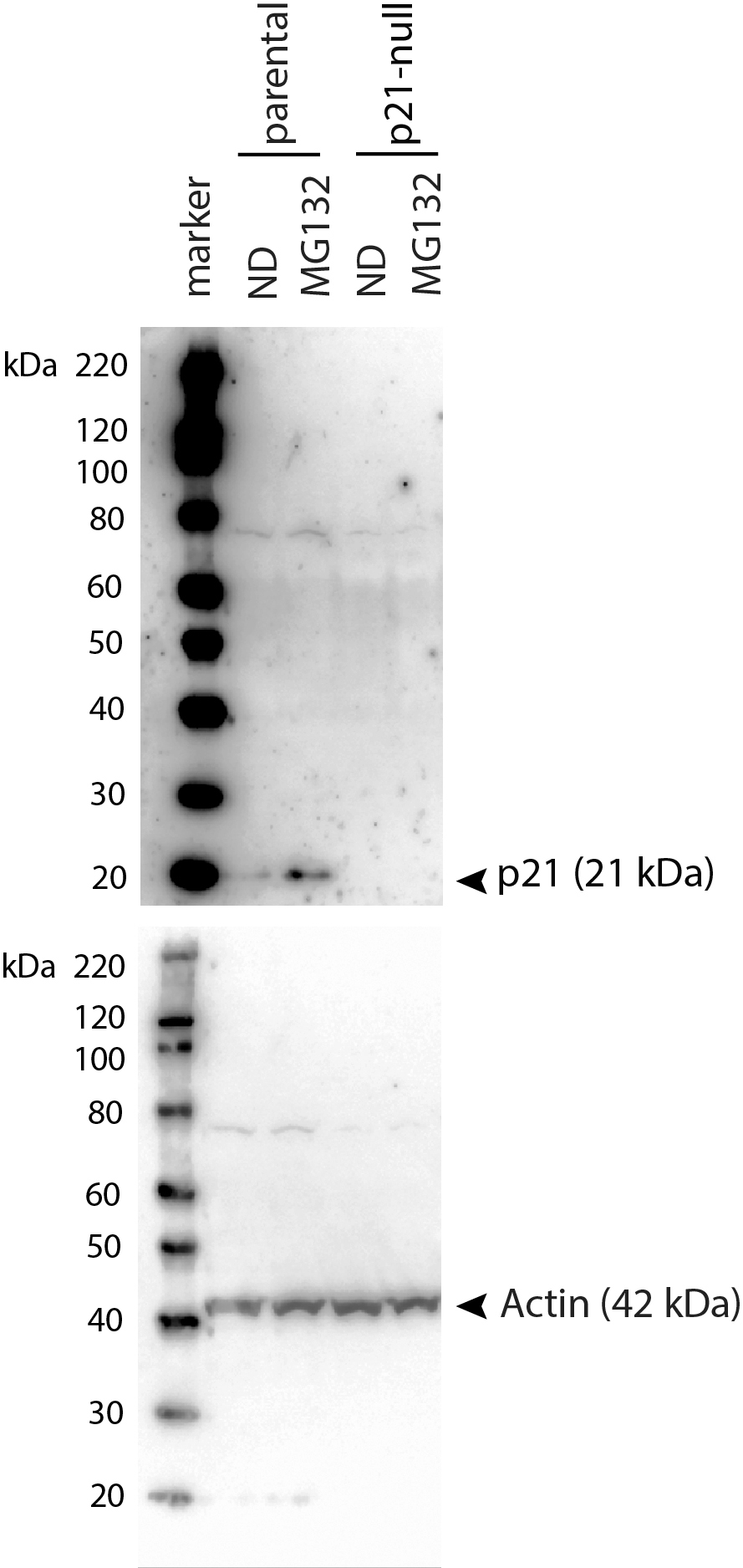

Supplement: Figure S3 — HCT116 and HCT116 p21 −/ − cells were treated with 10 µM MG132 for 8 h and immunoblot analysis was performed to confirm that CDKN1A-deleted cells didn’t express p21WAF1. [file peerj-11-16683-s003.jpg]

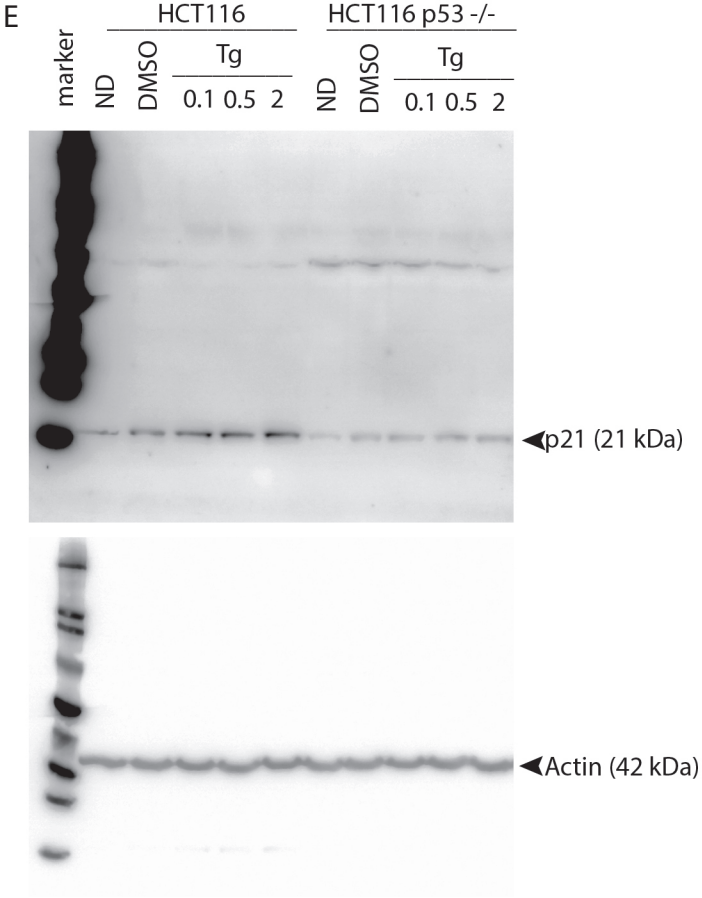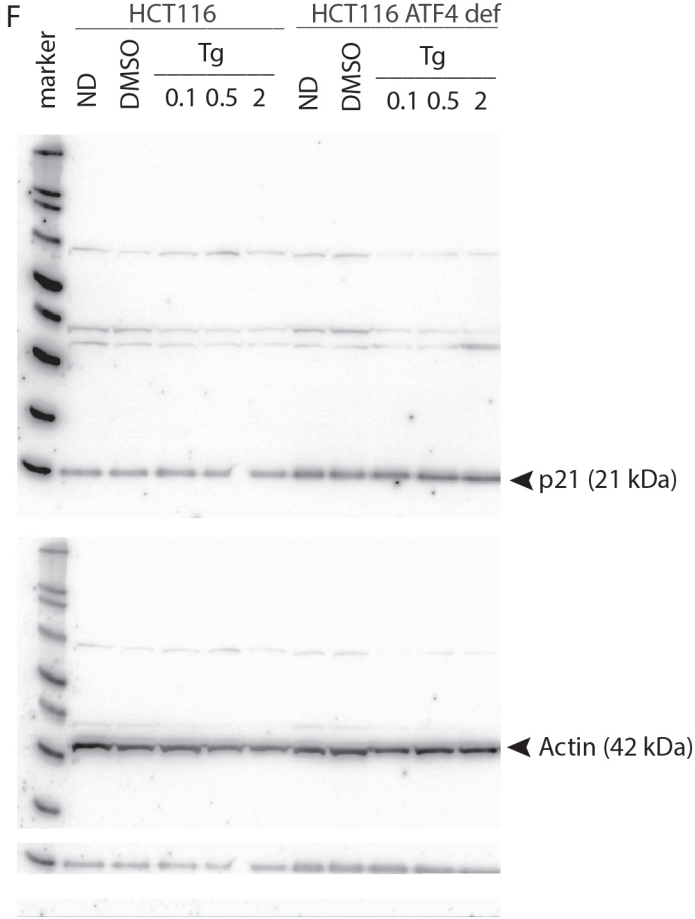

Supplement: Supplemental Information 5 — HCT116, HCT116 p53-/-, and HCT116 ATF4 def cells were treated with 0.1, 0.5 and 2 µM Tg for 8 h. Protein was extracted and immunoblot analysis of p21WAF1protein levels was completed. Immunoblots presented are representative of three biologically independent experiments. [file peerj-11-16683-s005.pdf]
