# Supplementary material for: Cyclin-dependent kinase inhibitor 1 plays a more prominent role than activating transcription factor 4 or the p53 tumour suppressor in thapsigargin-induced G1 arrest
Source: PeerJ. 2023 Dec 18;11:e16683. doi: 10.7717/peerj.16683 (PMC10734451; doi:10.7717/peerj.16683)
Supplement: Figure S4 — HCT116, HCT116 p53-/-, and HCT116 ATF4 def cells were treated with 0.1, 0.5 and 2 µM Tg for 8 h. Protein was extracted and immunoblot analysis of p21WAF1 protein levels was completed. Immunoblots presented are representative of three biologically independent experiments. [file peerj-11-16683-s004.pdf]

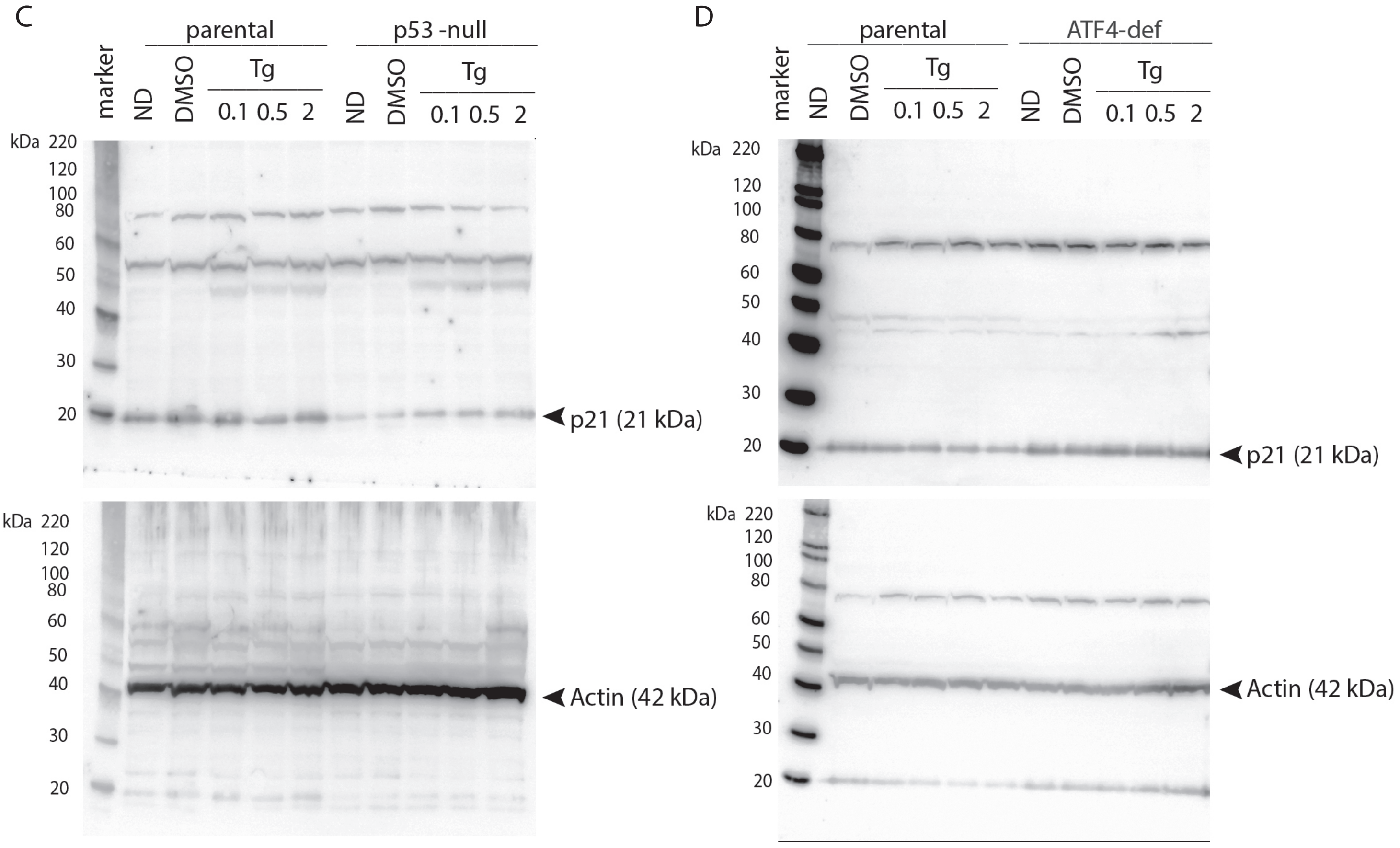

C
parental
p53 -null
marker
ND
DMSO
Tg
0.1 0.5 2
kDa 220
120
100
80
60
50
40
30
20
p21 (21 kDa)
Actin (42 kDa)
D
parental
ATF4-def
